# Effect of Neighborhood and Individual-Level Socioeconomic Factors on Colorectal Cancer Screening Adherence

**DOI:** 10.3390/ijerph18094398

**Published:** 2021-04-21

**Authors:** Kiara N. Mayhand, Elizabeth A. Handorf, Angel G. Ortiz, Evelyn T. Gonzalez, Amie Devlin, Kristen A. Sorice, Nestor Esnaola, Susan Fisher, Shannon M. Lynch

**Affiliations:** 1 Cancer Prevention and Control Program, Fox Chase Cancer Center, Philadelphia, PA 19111, USA; mayhank1@tcnj.edu (K.N.M.); angelgortiz1@gmail.com (A.G.O.); Kristen.Sorice@fccc.edu (K.A.S.); Susan.Fisher@tuhs.temple.edu (S.F.); 2 Department of Public Health, The College of New Jersey, Ewing, NJ 08618, USA; 3 Fox Chase Cancer Center, Population Studies and Biostatistics Facility, Philadelphia, PA 19111, USA; Elizabeth.Handorf@fccc.edu; 4 Office of Community Outreach & Engagement, Fox Chase Cancer Center, Philadelphia, PA 19111, USA; Evelyn.Gonzalez@fccc.edu; 5 Department of Clinical Sciences, Temple University, Philadelphia, PA 19140, USA; Amie.Devlin@drexel.edu; 6 Department of Surgery Houston Methodist Hospital Houston Methodist Weill Cornell Medical College, Houston, TX 77030, USA; nfesnaola@houstonmethodist.org

**Keywords:** colorectal cancer, screening adherence, socioeconomic status, neighborhood factors, disparities

## Abstract

Despite the effectiveness of screenings in reducing colorectal cancer (CRC) mortality, ~25% of US adults do not adhere to screening guidelines. Prior studies associate socioeconomic status (SES) with low screening adherence and suggest that neighborhood deprivation can influence CRC outcomes. We comprehensively investigated the effect of neighborhood SES circumstances (nSES), individual SES, and race/ethnicity on adherence to CRC screening in a multiethnic cross-sectional study. Participant surveys assessing 32 individual-level socioeconomic and healthcare access measures were administered from 2017 to 2018. Participant data were joined with nine nSES measures from the US Census at the census tract level. Univariate, LASSO, and multivariable mixed-effect logistic regression models were used for variable reduction and evaluation of associations. The total study population included 526 participants aged 50–85; 29% of participants were non-adherent. In the final multivariable model, age (*p* = 0.02) and Non-Hispanic Black race (*p* = 0.02) were associated with higher odds of adherence. Factors associated with lower adherence were home rental (vs. ownership) (*p* = 0.003), perception of low healthcare quality (*p* = 0.006), no routine checkup within two years (*p* = 0.002), perceived discrimination (*p* = 0.02), and nSES deprivation (*p* = 0.02). After comprehensive variable methods were applied, socioeconomic indicators at the neighborhood and individual level were found to contribute to low CRC screening adherence.

## 1. Introduction

Despite being largely preventable through screening assessments and having a 90% survival rate with early detection [1], colorectal cancer is the third leading cause of cancer-related deaths for both men and women in the United States (US) [2]. Colorectal cancer occurs when polyps, or abnormal growths, form in the colon or rectum. Based on the 2013–2017 national statistics, the age-adjusted mortality rate of colorectal cancer in the US is 13.9 per 100,000. Racial disparities with colorectal cancer mortality exist [3]. Non-Hispanic Black (NHB) men (23.2 per 100,000) and women (15.2 per 100,000) experience the highest colorectal cancer mortality rates compared to all other races/ethnicities [4]. Trends are similar in New Jersey (NJ) and Philadelphia, Pennsylvania (PA), the geographical focus of this study. The colorectal cancer mortality rates for NJ and Philadelphia are 14 and 17 per 100,000, with the highest mortality rates seen among NHB men (22.3 and 24.2 per 100,000) and NHB women (13.6 and 15.4 per 100,000) respectively [4]. 

Colorectal cancer can be prevented through timely screening and early detection/removal of pre-cancerous polyps [3,5]. There are multiple methods of preventative screening for colorectal cancer: colonoscopy, sigmoidoscopy, and guaiac-based fecal occult blood tests (gFOBT) [6,7]. As part of its cancer screening guidelines, the American Cancer Society (ACS) previously recommended that average-risk patients begin colorectal cancer screening with one of the aforementioned methods beginning at age 50, and to continue to adhere to the appropriate screening time intervals until the age of 75 [8]. Given the rise in colorectal cancer incidence among young adults, professional organizations, including the United States Preventive Services Task Force, have recently drafted revised guidelines to recommend that screening begin at the age of 45 [8,9]. In consultation with their physician, high-risk patients (e.g., those with a personal or familial history of colorectal cancer and those who have genetic syndromes or inflammatory bowel diseases) [10] are advised to undergo a comprehensive risk assessment and receive a colonoscopy annually [8]. Despite the effectiveness of adherence to timely screenings in reducing colorectal cancer mortality, about 25% of US adults do not adhere to screening guidelines [2]. 

Previous studies have identified specific individual-level risk factors and barriers associated with low rates of screening adherence among the general, average-risk US population. Individual demographic and socioeconomic factors such as education, income, social support, and access to and utilization of quality healthcare [11] were found to be associated with adherence to recommended colorectal cancer screenings [12,13]. Other barriers to screening fall under overarching contextual themes such as cancer beliefs (e.g., fatalism or fear of receiving a cancer diagnosis), perceptions of cancer risk (e.g., belief that the risk of having cancer is low), and lack of knowledge (e.g., awareness of cancer screening guidelines) [11]. 

In addition to an individual’s socioeconomic status (e.g., education, income, literacy), healthcare access (quality care, having insurance, and transportation) and beliefs (related to cancer risk, fatalism, and medical mistrust); the neighborhood environment in which a person lives may also influence cancer outcomes [14]. Previous studies have found associations between low neighborhood socioeconomic environments (nSES; often defined in terms of the education, employment, income, poverty of an area) and colorectal cancer mortality [15]. These associations remained even after adjustment for individual socioeconomic measures, suggesting that nSES measures can exert independent effects on cancer outcomes [16,17,18]. Further, the Kurani and colleagues’ study on screening rates in the US Midwest found that individuals living in the most deprived neighborhoods were about 50% less likely to be adherent to screening compared to those residing in the least deprived areas [19]. However, the aforementioned study did not include additional individual-level measures, such as cancer beliefs or SES measures, typically associated with screening adherence. Given that it is possible that individual-level factors compounded with nSES measures may provide insight into low screening adherence among populations, more comprehensive investigations are warranted. 

To our knowledge, few studies have comprehensively investigated the impact of both neighborhood and individual-level factors on colorectal cancer screening adherence. A number of multilevel conceptual frameworks in cancer exist that suggest that neighborhood or community, along with individual and interpersonal factors, work together to influence cancer outcomes and related behaviors [20,21]. Most studies of neighborhood and colorectal cancer have focused on incidence and mortality, and less on screening, and each often selects a different measure to represent neighborhood deprivation or nSES [15,19,22,23]. Further, few studies have investigated the impact of additional individual-level healthcare system factors and sociocultural measures beyond socioeconomic measures such as insurance status, income, or education to include assessments of financial or food insecurity and perceived discrimination in society and health care [18]. These indicators have been assessed in other disease settings, such as cardiovascular disease or diabetes, and for other cancer sites (such as breast, prostate, and cervix) [24,25,26]. Financial insecurity has been assessed in colorectal cancer patients’ post-diagnosis/treatment and was found to have a significant association with survivorship and lower quality of life but has yet to be evaluated in the preventative context as a potential indicator for low colorectal cancer screening adherence [27,28]. Thus, under the guidance of the multilevel National Institute on Minority Health and Health Disparities (NIMHHD) Research Framework [20], the goal of this study was to expand and comprehensively explore which neighborhood and individual-level factors influence colorectal cancer screening adherence in a multiethnic cohort from the Philadelphia area. Significant associations found in this study will be beneficial in identifying characteristics of low adherence populations to target for colorectal cancer screening and educational interventions. 

## 2. Materials and Methods

### 2.1. Study Population

This study sample was identified from the 1000 participants in the Population Health Assessment in Cancer Center Catchment Areas (PHA) study (IRB#17-8005) [29], a National Cancer Institute effort to support survey development using nationally validated instruments [30], data collection, and data linkage efforts that could help define and describe underserved cancer center populations across the US [29]. For this effort, a variety of sampling approaches were supported to capture often-understudied populations [29]. At the center of the Fox Chase Cancer Center (FCCC) catchment area of southeastern Pennsylvania and southern New Jersey is Philadelphia. PHA recruitment was planned to include at least 500 subjects from the diverse, underserved, and socioeconomically disadvantaged population of Philadelphia. These community residents were recruited through *Temple Health: Block-By-Block* (THB^3^), a population-based cohort study focused on community health improvement [31]. Recruitment was supplemented via snowball sampling and sporadic convenience sampling at neighborhood venues and community events. An additional 500 subjects were recruited via convenience sampling by the FCCC Office of Community Outreach (OCO). Specifically, OCO recruitment strategies included re-engagement of previous research participants (who previously agreed to be re-contacted regarding future studies), as well as distribution of recruitment flyers at community events with existing community partners that include federally qualified health clinics, faith-based organizations, housing agencies, etc. Data were collected between July 2017 and May 2018 with 75% of the final study sample meeting at least one of the following criteria: residence in a medically underserved area (as defined by the Health Resources and Services Administration) [32]; residence in a “low education” ZIP code (i.e., <79% of residents graduated from high school); residence in a “low income” ZIP code (i.e., median income < $38,000); coverage by subsidized health insurance (i.e., Medicaid or state-subsidized plans for low-income adults); or lack of health insurance.

Study eligibility included being 18 years of age or older, able to understand English or Spanish, residing in the FCCC catchment area, and willing to provide a local address. Patient address information was geocoded to the census tract level and assigned a Federal Information Processing Standard (FIPS) code to allow for linkage of participant data to the census-tract neighborhood and neighborhood-level variables. We limited our analysis to participants between the ages of 50 and 85 based upon colorectal cancer screening guidelines and because data collection procedures only asked participants aged 50 or older to respond to cancer screening questions. We excluded from analysis individuals missing data on key variables, including race (*n* = 3), home rental/ownership (*n* = 1), perceived discrimination (*n* = 5), and last visit to a doctor for a routine check-up (*n* = 1), as well as those for whom screening adherence could not be determined (*n* = 8). A total of 526 participants were included in the study. This cross-sectional study was approved by the institutional review boards at Temple University and FCCC.

### 2.2. Outcome Variable

The primary outcome in this analysis was colorectal cancer screening adherence status (adherent vs. non-adherent), defined based on guidelines from the ACS [8], the National Comprehensive Cancer Network (NCCN) [33], and the United States Preventive Services Task Force (USPSTF) [34] at the time of study enrollment. We coded adherence using survey responses regarding receipt of colonoscopy, sigmoidoscopy, and guaiac-based fecal occult blood stool test (gFOBT). Participants were adherent if they were average risk (no personal history of colon cancer) and met one or more of the following criteria:

1. Age 50 to 85 and had a colonoscopy within the last 10 years;
2. Age 50 to 80 and had a sigmoidoscopy within the last five years;
3. Age 50 to 76 and had a gFOBT in the last year.

Participants were additionally coded as adherent if they had a history of colorectal cancer and received a colonoscopy within the past year (3 of 6 participants with a colorectal cancer history were coded as adherent). Individuals not meeting these requirements were labeled non-adherent (Table S1A, outcome definitions).

### 2.3. Individual-Level Variables

The PHA survey collected data across multiple key constructs based on multilevel conceptual frameworks suggesting that behavioral, social, and physiologic factors impact cancer outcomes [29,35]. This original framework was further expanded by the National Institute on Minority Health and Health Disparities Research Framework (NIMHHD) [20], which postulates that behaviors, physical and built environment, socioeconomic-cultural environment, and healthcare system factors can influence society, communities, families (interpersonal) and ultimately individuals. Thus, we chose to assess individual-level factors from the following constructs based on this framework and previous findings suggesting their impact on cancer health disparities and colorectal cancer adherence: sociocultural environment measures including sociodemographics [3,12,13], economic measures [12,27,28], perceived discrimination [25,26,36,37,38], cancer knowledge/beliefs [11], social support [11], cancer-related risk behaviors [11]; and healthcare system measures including health literacy [39,40,41,42], and healthcare access [12] (see Table 1 and Table S1B).

### 2.4. Physical/Neighborhood Variables (nSES Variables)

In addition to participant-level factors, our study accounted for the physical and built environment under the NIMHHD Research Framework [20], using measures from previous research found to be associated with cancer mortality or screening adherence [18,19]. Neighborhood data were obtained from the United States Census American Community Survey (2014–2018) at the census tract level since these were the most recent five-year estimates that overlapped with our recruitment period at the time of analysis. We did compare geocode estimates between the recently released (December 2020) ACS 2015–2019, and estimates were not significantly different than the ACS 2014–2018 measures used in this study. Physical/Neighborhood-level variables were linked to participant data using a Federal Information Processing Standard (FIPS) code at the census tract level in R (R Core Team, Vienna, Austria) [43]. Physical/Neighborhood variables analyzed in this study include: (1) Neighborhood Stability; (2) Language Skills; (3) Household Isolation; (4) Household Income; (5) Crowding; (6) Transportation; (7) Multiple Index of Concentration at the Extremes (ICE) measures [16,44,45,46]; and (8) Yost deprivation index [47,48] (see Table 1 and Table S1B).

### 2.5. Statistical Analysis

Continuous physical/neighborhood and individual-level variables were summarized by means and medians; categorical variables were summarized by percentages. Next, we systematically evaluated the independent association between each individual- and neighborhood-level variable with the binary outcome (colorectal cancer screening adherence). Specifically, we first ran univariate logistic regression models [49] to identify individual-level and neighborhood-level variables significantly associated with colorectal cancer screening adherence at a Wald *p*-value < 0.20 in the total study population (univariate *p*-values reported in Table 1) and individually for NHB, Non-Hispanic White (NHW), and Hispanic participants (univariate *p*-values reported in Table S1C). Further, for univariate analysis, missing covariate data were generally handled by taking a complete case analysis for the specific model under consideration. To maximize the sample size, informative observations were included wherever possible. Not sure/refused responses were combined as the numbers of participants with these responses were small. Additionally, sensitivity analyses were performed in which subjects with any missing covariates (including not sure/refused responses) were excluded and results did not change. As an additional variable reduction step, we applied LASSO machine learning approaches [50] to account for the high degree of correlation within individual and neighborhood variables (Table S1D). Covariates with values that did not shrink to zero in LASSO were considered relevant predictors of the outcome.

Variables that were significant in the univariate analysis at a *p*-value < 0.20 and that were identified as predictors in LASSO (non-zero values) were marked with an asterisk (*) in Table 1. Although not marked with an asterisk, given previously identified associations between gender and screening behaviors [51,52,53] and differences in sampling approaches by center in our study, sex and center were retained in the development of multivariate models given potential for confounding. Of note, multivariable models did not include 3 specific knowledge/belief items which, by the survey design, were only asked of a subset of participants without a cancer history. These 3 variables (Compared to other people your age, how likely do you think you are to get cancer in your lifetime; I’d rather not know my chance of getting cancer; and How worried are you about getting cancer) relate to getting cancer and were considered not applicable and potentially burdensome to patients who might have past cancer history. Next, we refined the model via backward elimination in a series of mixed-effect multivariable logistic regression models which accounted for potential neighborhood clustering effects [54] (Table S1E). A likelihood ratio test was run after each backward regression to determine the best model fit and the final model [55]. Odds ratios representing screening adherence, 95% confidence intervals (CI), and *p*-values from the final model in the total study population are reported in Table 2, with *p*-values < 0.05 being considered statistically significant. Univariate and LASSO approaches were also applied in race-stratified groups (Table S1C,D) to evaluate if findings differed by race. However, given small sample sizes when stratifying by race group, multivariable models using the total study population serve as the main findings.

## 3. Results

### 3.1. Univariate Analysis

Study participants (*n* = 526) were between the ages of 50 and 85 (mean = 62, standard deviation (SD) = 8.4); 35% were male and 65% were female. Fifty-seven percent of participants were NHB, 27% were NHW, and 13% were Hispanic. Twenty-nine percent of the participants were non-adherent to colorectal cancer screening. While many of the sociodemographic, economic, and healthcare access variables were significant at a *p*-value < 0.05, fewer variables also replicated in the LASSO analysis, (which accounts for multicollinearity) and were identified for subsequent multivariable regression (Table 1). 

Non-adherence was significantly associated with age and differed by race/ethnicity. Non-adherence was more prevalent among participants who rented their home compared to owning (*p* < 0.001); were of low income (*p* = 0.002); needed to see a physician but were unable due to cost (*p* = 0.01); had not visited a doctor for a routine checkup in over two years (*p* < 0.001); rated the quality of their health care as fair or poor (*p* < 0.001); generally felt they were treated worse than people of other races (*p* = 0.003); disagreed that a person’s behavior or lifestyle contributes to cancer outcomes (*p* = 0.009); and who indicated that they would rather not know their chances of receiving a cancer diagnosis (*p* = 0.024). However, due to the high degree of missing data, we excluded from analysis the variable which indicated whether a person would rather not know their chances of receiving a cancer diagnosis. Although not significant, it is also worth noting that the majority (57%) of non-adherent participants incorrectly identified the correct age to begin colorectal cancer screening. 

Non-adherent participants were more likely to live in neighborhoods of concentrated poverty (ICE-Income, *p* = 0.002), low SES conditions (Yost index, *p* < 0.001) and were less likely to have access to a vehicle (*p* < 0.001) (Table 1).

### 3.2. Multivariable Analysis

Variables identified as significant in the univariate analysis and LASSO were applied in a full multivariable model (Table S1E—first model). Backward regression and likelihood ratio test comparisons were then applied to identify the best model fit (Table 2—final model). Covariates that remained significant in the final model for the total population are described here. In multivariable-adjusted models, increasing age was associated with being adherent (OR = 1.04, 95% CI 1.01–1.07, *p* = 0.02). NHB participants were significantly more likely to report adherence to colorectal cancer screening (OR = 2.72, 95% CI = 1.32–5.59, *p* = 0.002) than NHW participants. Hispanic participants were not significantly different than NHW participants. Odds of colorectal cancer screening adherence were significantly lower for those who rented their home (OR = 0.50, 95% CI = 0.31–0.82, *p* = 0.003) or had not received a routine checkup in more than 2 years (OR = 0.17, 95% CI = 0.06–0.48, *p* = 0.002). Individuals who reported the quality of health care they received as excellent/very good had higher odds of adherence than those who rated their care as good (OR = 0.51, 95% CI = 0.33–0.80, *p* = 0.002) or fair/poor (OR = 0.35, 95% CI = 0.18–0.66 *p* < 0.001). Interestingly, when asked about discrimination, individuals who thought they were treated better (OR = 0.45, 95% CI = 0.24–0.83 *p* = 0.01) or worse (0.44, 95% CI = 0.23–0.83 *p* = 0.01) than other racial/ethnic groups had lower adherence than those who reported that they were treated the same as other races. There was also a significant dose–response association between nSES and adherence. Specifically, participants living in low SES neighborhoods (as measured by the Yost Index Quantiles) had the lowest odds of adherence (Lowest SES neighborhood OR = 0.28, 95% CI = 0.1–0.73; *p* = 0.01) compared to those living in higher SES neighborhoods. 

## 4. Discussion

We have comprehensively evaluated and identified individual- and neighborhood-level variables (both new and previously identified) that could contribute to colorectal cancer screening adherence in a multiethnic, underserved study population from the Philadelphia area. The proportion of participants reporting non-adherence to colorectal cancer screening in our study (29%) is similar to national statistics (25%) [2]. We also found that adherence to colorectal cancer screening guidelines was higher among NHB compared to NHW participants. At first, this seemed surprising based on univariate results; however, our findings are consistent with previous population-based studies in Philadelphia, which also report significantly higher colorectal cancer screening rates for NHB participants compared to NHW and Hispanic participants in models that include adjustments for age and sex [56]. Similar to prior studies and in line with hypotheses in the NIMHHD framework [20], we also found associations between measures of healthcare access, nSES, and screening adherence. Specifically, not routinely seeing a doctor for more than 2 years [12,13], and lower quality of care ratings were associated with lower odds of adhering to colorectal cancer screening [12,13]. Similar to prior work, we also found that living in lower SES areas was associated with lower odds of adhering to colorectal cancer screening [19]; however, here we demonstrate that this association remained even in the presence of adjustments for a more comprehensive list of individual-level SES variables. 

Most prior studies have assessed and identified economic measures, such as income, employment, and insurance status as significant factors affecting cancer screening adherence [12]. As such, we incorporated additional economic measures including financial and food insecurity questions, anticipating that economic variables would play a role in adherence. However, economic factors were not significant in our final model. Instead, we identified new variables associated with screening adherence, namely renting vs. owning a home and perceived discrimination. Renting a home is a socioeconomic measure that has been studied in relation to cancer incidence studies [57], but few, if any, have evaluated this variable in the context of screening adherence. In particular, prior literature has identified renting a home as an indicator of housing instability, overcrowding, and food insecurity [58,59]; whereas owning a home is often an indicator of financial security, higher income, and living in more affluent neighborhoods with greater access to quality health services. Given the associations with economic indicators, it is possible that homeownership may serve as an additional economic measure of SES for health disparities research and/or as a surrogate for the accessibility of healthcare services, including screening adherence.

Few studies have assessed the impact of perceived discrimination on cancer screening outcomes. In prior literature, individuals who reported experiencing racial discrimination in healthcare settings were less likely to be up to date with screening adherence for breast and colorectal cancer, specifically [60]. In our study, we found that participants who thought, in general, that they were treated worse (indicating feelings of racial discrimination) or better than other race groups (indicating feelings of racial privilege) had lower odds of adherence to colorectal cancer screening guidelines. When we looked specifically at the response options to this question by race, we noted potential interactions. The vast majority (94%) of NHW participants felt they were treated the same as or better than other race groups compared to 86% of Hispanics and 77% of NHB participants. However, when looking specifically at adherence within race groups, NHW and Hispanic participants did not have large differences in the percentage of non-adherent participants who reported being treated worse than other race groups, whereas 27.5% of non-adherent NHB participants reported being treated worse than other races compared to 13.4% of adherent NHB participants. Recent studies present findings of associations between the perception of racial discrimination and increased likelihood to forego and underutilize care [37]. While it is unlikely that individual perception of general racial discrimination is responsible for all health disparities in preventative services, such as cancer screenings [25], these patterns reflect prior studies highlighting the presence of systemic racism and its intersectional role with other individual and neighborhood SES factors to impact health outcomes, such as screening adherence, particularly with NHB participants [61,62]. Thus, investigations into discrimination beyond the NIMHHD framework, perhaps based on the discrimination-focused ecosocial framework proposed by Krieger [63], are warranted in future cancer studies.

There are some study limitations to note. Our study is cross-sectional and collected survey data at one point in time from a non-random study sample, thus limiting our ability to evaluate causal relationships. We utilized a nonprobability sampling approach to target participants from underserved populations, and this approach could introduce selection bias, particularly because the sample may not be fully representative of population-based socioeconomic or race/ethnic groups. More females than males participated in this study, which is a common observation in cross-sectional and interventional cancer research studies [64]. This could be important, particularly in colorectal cancer, because there are differences in colorectal cancer mortality rates in Philadelphia and nationally by both race/ethnicity and gender [56,65]. However, when stratifying analyses by sex in our study, results did not differ between men and women. While our analysis included many measures believed to be associated with cancer disparities based on multilevel frameworks, we were limited to measures selected as part of the funded PHA supplement. It is possible additional covariates not included in this analysis, for instance segregation or healthcare screening access (i.e., related to geographic density and distance), may also provide additional insights. Further, our variable for CRC screening adherence is reliant on self-reported participant data, which are subject to recall bias and may not be as accurate as medical records. Due to the design of the PHA survey, some questions (i.e., cancer beliefs and knowledge) were only asked to participants who did not have a previous history of cancer, resulting in missing data. Thus, these variables were excluded from multivariate models, and only univariate results are presented for transparency and to provide crude estimates that may inform future studies. Inclusion of an indicator for missing data is generally not recommended [66] due to potential for bias; however, concerns related to bias introduced by missing data are likely minimal in our study given that results were similar when restricting our sample to participants without missing data. The United States Preventive Services Task Force recently released a draft of updated guidelines to recommend that colorectal cancer screening begin at age 45 [9]; however, in line with previous guidelines, the survey questions specific to colorectal cancer screening adherence were only administered to and analyzed for participants 50 years of age or older. Additionally, evaluation of adherence could be improved if we were able to determine the age at first colorectal cancer screening. Some of our variables were highly correlated with each other and it is possible that some findings could be due to chance; however, we attempted to minimize the impact of multicollinearity by using LASSO approaches. Confounding, interaction, and mediation effects likely also exist, particularly by race but also by measures of economic, sociodemographic, and perceived discrimination. This suggests that future studies with a larger, multiethnic study sample are needed to account for these effects and to determine if findings might change/vary within race/ethnic groups. We did conduct univariate and LASSO analyses by race/ethnicity, and findings in the total study population were generally also found in at least one other race/ethnic group (Table S1C).

## 5. Conclusions

After a systematic and rigorous methodologic assessment, we identified that homeownership, perceived quality of medical care, utilization of routine doctor’s visits, perceived discrimination, and nSES factors were associated with colorectal cancer screening adherence. While these findings can be used to identify individuals and communities in need of targeted interventions locally, they also inform health disparities research more broadly. More specifically, under a Precision Public Health framework, we plan to utilize sources of surveillance data (e.g., age, nSES deprivation, race/ethnicity, and rented household information from the US Census) to more efficiently guide colorectal cancer screening outreach in our cancer center catchment [46]. For example, screening efforts that identify patients living in low nSES neighborhoods and who live in rented households may be more efficient than targeting a specific race/ethnic group. Further, findings at the individual level related to perceived quality of medical care and utilization of routine doctor’s visits are likely intertwined with and indicative of the perceived discrimination reported by participants in this study. Our preliminary findings suggest that perceived discrimination may impact colorectal cancer screening adherence for some race/ethnic groups. This finding suggests that future investigations into the role of perceived discrimination on cancer outcomes in larger, multiethnic samples is warranted. The comprehensive methodological assessments in this study are particularly timely, given that professional organizations are now calling for more comprehensive assessments in health disparities research that include standardized sets of socioeconomic variables at both individual and neighborhood levels [67]. Increased efforts to regulate how we comprehensively study health disparities will not only increase our understanding of the impact of health inequities on health outcomes but also further prioritize the need for cultural humility, tangible education and trainings, and policy interventions to reduce health disparities at the population level. 

## Figures and Tables

**Table 1:** Significant Baseline Characteristics of the Total Study Population Based on Univariate Logistic Regression Models.

Total Study Population (*n* = 526)				
	Non-Adherent	Adherent	Total	*p*-Value
	(*n* = 151)	(*n* = 375)	(*n* = 526)	
**Sociocultural Environment**				
***Socio-Demographics***				
**Age**				<0.0001 *
Mean (SD)	59.1 (7.92)	62.9 (8.39)	61.8 (8.43)	
Median (Min, Max)	57.0 (50.0, 84.0)	62.0 (50.0, 85.0)	60.5 (50.0, 85.0)	
**Sex**				0.30
Female	93 (61.6%)	249 (66.4%)	342 (65.0%)	
Male	58 (38.4%)	126 (33.6%)	184 (35.0%)	
**Race/Ethnicity**				0.107 *
Non-Hispanic White	34 (22.5%)	113 (30.1%)	147 (27.9%)	
Non-Hispanic Black	91 (60.3%)	217 (57.9%)	308 (58.6%)	
Hispanic	26 (17.2%)	45 (12.0%)	71 (13.5%)	
**Center**				0.08
Fox Chase Cancer Center	73 (48.3%)	213 (56.8%)	286 (54.4%)	
Temple University Hospital	78 (51.7%)	162 (43.2%)	240 (45.6%)	
**Education**				0.11
More than high school	80 (53.0%)	231 (61.6%)	311 (59.1%)	
High school	41 (27.2%)	96 (25.6%)	137 (26.0%)	
Less than high school	29 (19.2%)	48 (12.8%)	77 (14.6%)	
Missing	1 (0.7%)	0 (0%)	1 (0.2%)	
**Marital Status**				0.005
Married	35 (23.2%)	137 (36.5%)	172 (32.7%)	
Not Married	114 (75.5%)	238 (63.5%)	352 (66.9%)	
Missing	2 (1.3%)	0 (0%)	2 (0.4%)	
**Country of Birth**				0.21
USA	140 (92.7%)	358 (95.5%)	498 (94.7%)	
Foreign	11 (7.3%)	17 (4.5%)	28 (5.3%)	
**Home rental or ownership**				<0.001 *
Rent/Other	101 (66.9%)	155 (41.3%)	256 (48.7%)	
Own	50 (33.1%)	219 (58.4%)	269 (51.1%)	
Missing	0 (0%)	1 (0.3%)	1 (0.2%)	
***Economic variables***				
**Household Income**				0.001 *
Low (Less than $10,000 to under $20,000)	70 (46.4%)	118 (31.5%)	188 (35.7%)	
Mid ($20,000 to under $75,000)	58 (38.4%)	132 (35.2%)	190 (36.1%)	
High (above $75,000)	13 (8.6%)	89 (23.7%)	102 (19.4%)	
Not sure/Refused				
**How often in the past 12 months would you say you were worried or stressed about having enough money to pay your rent/mortgage (financial insecurity)?**				<0.001
Always/Usually	47 (31.1%)	69 (18.4%)	116 (22.1%)	
Sometimes	41 (27.2%)	81 (21.6%)	122 (23.2%)	
Never/Rarely	51 (33.8%)	208 (55.5%)	259 (49.2%)	
Not sure/Refused	12 (7.9%)	17 (4.5%)	29 (5.5%)	
**How often in the past 12 months would you say you were worried or stressed about having enough money to buy nutritious meals (food insecurity)?**				0.003
Always/Usually	34 (22.5%)	54 (14.4%)	88 (16.7%)	
Sometimes	73 (48.3%)	245 (65.3%)	318 (60.5%)	
Never/Rarely	42 (27.8%)	76 (20.3%)	118 (22.4%)	
Missing	2 (1.3%)	0 (0%)	2 (0.4%)	
**Which one of these phrases comes closest to your own feelings about your household’s income these days?**				0.01
Living comfortably on present income	36 (23.8%)	133 (35.5%)	169 (32.1%)	
Getting by on present income	70 (46.4%)	169 (45.1%)	239 (45.4%)	
Finding it very difficult on present income/Finding it difficult on present income	43 (28.5%)	70 (18.7%)	113 (21.5%)	
Missing	2 (1.3%)	3 (0.8%)	5 (1.0%)	
***Perceived Discrimination***				
**Within the past 12 months, do you feel you were treated worse than, the same as, or better than people of other races?**				0.003 *
Better than other races	19 (12.6%)	36 (9.6%)	55 (10.5%)	
Only encountered people of the same race/The same as other races	94 (62.3%)	288 (76.8%)	382 (72.6%)	
Worse than other races	29 (19.2%)	33 (8.8%)	62 (11.8%)	
Not sure/Refused	8 (5.3%)	14 (3.7%)	22 (4.2%)	
Missing	1 (0.7%)	4 (1.1%)	5 (1.0%)	
**Within the past 12 months, when seeking health care, do you feel your experiences were worse than, the same as or better than for people of other races?**				0.07
Better than other races	14 (9.3%)	37 (9.9%)	51 (9.7%)	
Only encountered people of the same race/The same as other races	106 (70.2%)	300 (80.0%)	406 (77.2%)	
Worse than other races	12 (7.9%)	23 (6.1%)	35 (6.7%)	
Not sure/Refused	11 (7.3%)	10 (2.7%)	21 (4.0%)	
Missing	8 (5.3%)	5 (1.3%)	13 (2.5%)	
***Cancer Knowledge/Beliefs***				
**When I think about cancer, I automatically think about death. Would you say you …?**				0.05
Strongly agree/Somewhat agree	79 (52.3%)	161 (42.9%)	240 (45.6%)	
Strongly disagree/Somewhat disagree	70 (46.4%)	210 (56.0%)	280 (53.2%)	
Not sure/Refused	2 (1.3%)	4 (1.1%)	6 (1.1%)	
**There’s not much you can do to lower your chances of getting cancer. Would you say you …?**				0.14
Strongly agree/Somewhat agree	59 (39.1%)	126 (33.6%)	185 (35.2%)	
Strongly disagree/Somewhat disagree	87 (57.6%)	244 (65.1%)	331 (62.9%)	
Not sure/Refused	5 (3.3%)	5 (1.3%)	10 (1.9%)	
**There are so many different recommendations about preventing cancer, it’s hard to know which ones to follow. Would you say you?**				0.60
Strongly agree/Somewhat agree	31 (20.5%)	88 (23.5%)	119 (22.6%)	
Somewhat disagree/Strongly disagree	113 (74.8%)	283 (75.5%)	396 (75.3%)	
Not sure/Refused	7 (4.6%)	4 (1.1%)	11 (2.1%)	
**Cancer is most often caused by a person’s behavior or lifestyle. Would you say you?**				0.009 *
Strongly agree/Somewhat agree	66 (43.7%)	218 (58.1%)	284 (54.0%)	
Somewhat disagree/Strongly disagree	75 (49.7%)	143 (38.1%)	218 (41.4%)	
Not sure/Refused	10 (6.6%)	14 (3.7%)	24 (4.6%)	
**Compared to other people your age, how likely do you think you are to get cancer in your lifetime?**				0.95
Very Likely/Likely	47 (31.1%)	99 (26.4%)	146 (27.8%)	
Neither unlikely nor likely	34 (22.5%)	83 (22.1%)	117 (22.2%)	
Very unlikely/Unlikely	39 (25.8%)	84 (22.4%)	123 (23.4%)	
Not sure/Refused	11 (7.3%)	24 (6.4%)	35 (6.7%)	
Not Asked	20 (13.2%)	85 (22.7%)	105 (20.0%)	
**I’d rather not know my chance of getting cancer**				0.024 *
Strongly agree/Somewhat agree	56 (37.1%)	100 (26.7%)	156 (29.7%)	
Strongly disagree/Somewhat disagree	68 (45.0%)	185 (49.3%)	253 (48.1%)	
Not sure/Refused	7 (4.6%)	5 (1.3%)	12 (2.3%)	
Not Asked	20 (13.2%)	85 (22.7%)	105 (20.0%)	
**How worried are you about getting cancer?**				0.39
Moderately/Extremely	30 (19.9%)	51 (13.6%)	81 (15.4%)	
Somewhat	74 (49.0%)	181 (48.3%)	255 (48.5%)	
Not at all/Slightly	24 (15.9%)	57 (15.2%)	81 (15.4%)	
Not Asked	23 (15.2%)	86 (22.9%)	109 (20.7%)	
**At what age do you think people should begin screening for colorectal cancer?**				0.22
Correct	58 (38.4%)	175 (46.7%)	233 (44.3%)	
Incorrect	86 (57.0%)	183 (48.8%)	269 (51.1%)	
Not sure/Refused	7 (4.6%)	17 (4.5%)	24 (4.6%)	
***Social Network Support***				
**How often do you get the social and emotional support you need?**				0.004
Always/Usually	85 (56.3%)	263 (70.1%)	348 (66.2%)	
Rarely/Never	23 (15.2%)	30 (8.0%)	53 (10.1%)	
Sometimes	43 (28.5%)	79 (21.1%)	122 (23.2%)	
Missing	0 (0%)	3 (0.8%)	3 (0.6%)	
**Cancer-Related Risk Behaviors**				
**Current Smoker**				0.001
No	103 (68.2%)	303 (80.8%)	406 (77.2%)	
Yes	48 (31.8%)	70 (18.7%)	118 (22.4%)	
Missing	0 (0%)	2 (0.5%)	2 (0.4%)	
**Alcohol Use**				0.91
No	83 (55.0%)	209 (55.7%)	292 (55.5%)	
Yes	67 (44.4%)	165 (44.0%)	232 (44.1%)	
Missing	1 (0.7%)	1 (0.3%)	2 (0.4%)	
**Diabetes**				0.74
No	106 (70.2%)	268 (71.5%)	374 (71.1%)	
Yes	45 (29.8%)	106 (28.3%)	151 (28.7%)	
Missing	0 (0%)	1 (0.3%)	1 (0.2%)	
**Body Mass Index (BMI)**				0.66
Mean (SD)	29.9 (7.67)	30.2 (6.93)	30.1 (7.14)	
Median (Min, Max)	28.3 (16.6, 54.9)	28.7 (16.3, 57.0)	28.7 (16.3, 57.0)	
Missing	3 (2.0%)	3 (0.8%)	6 (1.1%)	
**Healthcare System Measures**				
***Health Literacy***				
**How difficult is it for you to understand information that doctors, nurses, etc., tell you?**				0.33
Very Difficult/Somewhat difficult	29 (19.2%)	59 (15.7%)	88 (16.7%)	
Very easy/Somewhat easy	121 (80.1%)	315 (84.0%)	436 (82.9%)	
Missing	1 (0.7%)	1 (0.3%)	2 (0.4%)	
***Healthcare Access***				
**Insurance Type**				<0.001
Medicaid	57 (37.7%)	104 (27.7%)	161 (30.6%)	
Medicare/Veteran	37 (24.5%)	134 (35.7%)	171 (32.5%)	
Not Insured	20 (13.2%)	15 (4.0%)	35 (6.7%)	
Private	36 (23.8%)	118 (31.5%)	154 (29.3%)	
Missing	1 (0.7%)	4 (1.1%)	5 (1.0%)	
**In the past 12 months, was there a time when you needed to see a doctor but could not because of the cost?**				0.012 *
No	120 (79.5%)	331 (88.3%)	451 (85.7%)	
Yes	30 (19.9%)	43 (11.5%)	73 (13.9%)	
Missing	1 (0.7%)	1 (0.3%)	2 (0.4%)	
**About how long has it been since you last visited a doctor for a routine checkup?**				<0.001 *
More than 2 years ago	17 (11.3%)	7 (1.9%)	24 (4.6%)	
Within the past year/Within past 2 years (1 year but less than 2 years ago)	134 (88.7%)	367 (97.9%)	501 (95.2%)	
Missing	0 (0%)	1 (0.3%)	1 (0.2%)	
**Overall, how would you rate the quality of health care you received in the past 12 months?**				<0.001 *
Excellent/Very Good	67 (44.4%)	254 (67.7%)	321 (61.0%)	
Good	54 (35.8%)	95 (25.3%)	149 (28.3%)	
Fair/Poor	22 (14.6%)	20 (5.3%)	42 (8.0%)	
Not sure/Refused	8 (5.3%)	6 (1.6%)	14 (2.7%)	
**Physical/Neighborhood-level variables**				
**Living in same house as 1 year ago**				0.13
Mean (SD)	0.870 (0.0720)	0.880 (0.0634)	0.877 (0.0660)	
Median (Min, Max)	0.884 (0.607, 0.982)	0.888 (0.549, 0.984)	0.888 (0.549, 0.984)	
**% Poor English**				0.49
Mean (SD)	0.0749 (0.0927)	0.0691 (0.0857)	0.0708 (0.0877)	
Median (Min, Max)	0.0359 (0, 0.375)	0.0353 (0, 0.422)	0.0353 (0, 0.422)	
**Household Isolation**				0.32
Mean (SD)	0.0550 (0.0841)	0.0479 (0.0702)	0.0499 (0.0744)	
Median (Min, Max)	0.0187 (0, 0.393)	0.0187 (0, 0.393)	0.0187 (0, 0.393)	
**ICE-Income (Quartiles)**				0.002 *
1 Concentrated Poverty	108 (71.5%)	208 (55.5%)	316 (60.1%)	
2	18 (11.9%)	40 (10.7%)	58 (11.0%)	
3	10 (6.6%)	53 (14.1%)	63 (12.0%)	
4 Concentrated Affluence	15 (9.9%)	74 (19.7%)	89 (16.9%)	
**ICE Race + Income (Quartiles)**				0.005
1 Concentrated Poverty of NHB	120 (79.5%)	241 (64.3%)	361 (68.6%)	
2	13 (8.6%)	39 (10.4%)	52 (9.9%)	
3	8 (5.3%)	29 (7.7%)	37 (7.0%)	
4 Concentration Affluence of NHW	10 (6.6%)	66 (17.6%)	76 (14.4%)	
**Yost Index (Quintiles)**				<0.001 *
1 Low SES	98 (64.9%)	175 (46.7%)	273 (51.9%)	
2	24 (15.9%)	60 (16.0%)	84 (16.0%)	
3	11 (7.3%)	29 (7.7%)	40 (7.6%)	
4	5 (3.3%)	44 (11.7%)	49 (9.3%)	
5 High SES	10 (6.6%)	58 (15.5%)	68 (12.9%)	
Missing	3 (2.0%)	9 (2.4%)	12 (2.3%)	
**Median-Household Income**				<0.001 *
Mean (SD)	37,700 (25,000)	48,500 (31,200)	45,400 (29,900)	
Median (Min, Max)	27,600 (14,000, 123,000)	35,800 (11,400, 155,000)	32,700 (11,400, 155,000)	
**% Overcrowding**				0.09
% Overcrowding High	81 (53.6%)	172 (45.9%)	253 (48.1%)	
% Overcrowding Low	70 (46.4%)	203 (54.1%)	273 (51.9%)	
**% with Access to Transportation (1 or more vehicles)**				<0.001 *
Mean (SD)	0.649 (0.198)	0.719 (0.204)	0.699 (0.205)	
Median (Min, Max)	0.574 (0.343, 0.995)	0.719 (0.357, 1.00)	0.666 (0.343, 1.00)	

* denotes variables that are significant in the univariate analysis at *p* < 0.2 and identified as a predictor by LASSO (Table S1D).

**Table 2:** Final Multivariate Analysis: Total Population (*n* = 507).

	Odds Ratio Estimate	Lower CI	Upper CI	ProbZ	Overall *p*-Value
**Sociocultural Environment**					
***Socio-Demographics***					
**Age**	1.04	1.01	1.07	0.02	0.02
**Sex**					0.52
Female	0.86	0.55	1.36	0.52	
Male	ref.				
**Race**					0.02
Non-Hispanic Black	2.72	1.32	5.59	0.01	
Hispanic	1.58	0.73	3.39	0.24	
Non-Hispanic White	ref.				
**Center**					0.13
Temple University Hospital	0.68	0.41	1.11	0.12	
Fox Chase Cancer Center	ref.				
**Home rental or ownership**					0.003
Rent/Other	0.50	0.31	0.82	0.01	
Own	ref.				
***Perceived Discrimination***					
**Within the past 12 months, do you feel you were treated worse than, the same as, or better than people of other races?**					0.02
Better than other races	0.45	0.24	0.83	0.01	
Only encountered people of the same race/The same as other races	ref.				
Worse than other races	0.44	0.23	0.83	0.01	
Not sure/Refused	0.62	0.19	2.00	0.42	
***Cancer Knowledge/Beliefs***					
**Cancer is most often caused by a person’s behavior or lifestyle. Would you say you?**					0.07
Somewhat disagree/Strongly disagree	0.65	0.42	1.03	0.07	
Strongly agree/Somewhat agree	ref.				
Not sure/Refused	0.38	0.14	1.02	0.05	
**Healthcare System Measures**					
***Healthcare Access***					
**About how long has it been since you last visited a doctor for a routine checkup?**					0.002
More than 2 years ago	0.17	0.06	0.48	0.001	
Within the past year/Within past 2 years (1 year but less than 2 years ago)	ref.				
**Overall, how would you rate the quality of health care you received in the past 12 months?**					0.006
Excellent/Very Good	ref.				
Good	0.51	0.33	0.80	0.003	
Fair/Poor	0.35	0.18	0.66	0.001	
Not sure/Refused	0.28	0.08	1.00	0.05	
**Physical/Neighborhood-level variables**					
**Yost Index (Quintiles)**					0.02
1 Low SES	0.28	0.11	0.71	0.01	
2	0.31	0.13	0.77	0.01	
3	0.34	0.12	0.96	0.04	
4	1.09	0.31	3.83	0.89	
5 High SES	ref.				

## Data Availability

The data presented in this study are available upon request from the corresponding author. The data are not publicly available due to the consent provided by participants on the use of confidential data.

## Supplementary Materials

The following are available online https://www.mdpi.com/article/10.3390/ijerph18094398/s1; Table S1A: Outcome Definitions; Table S1B: Description of Individual and Neighborhood-Level Variables; Table S1C: Significant Baseline Characteristics by Race/Ethnicity Based on Univariate Regression Models; Table S1D: LASSO Results; Table S1E: First Multivariable Analysis Model Results.
